# Psychological Resilience, Experimentally Manipulated Social Status, and Dietary Intake among Adolescents

**DOI:** 10.3390/nu13030806

**Published:** 2021-03-01

**Authors:** Victoria Guazzelli Williamson, Alexandra M. Lee, Darci Miller, Tianyao Huo, Jon K. Maner, Michelle Cardel

**Affiliations:** 1 Department of Psychology, University of Oregon, Eugene, OR 97403, USA; 2 Department of Health Outcomes and Biomedical Informatics, University of Florida, Gainesville, FL 32611, USA; alexlee22@ufl.edu (A.M.L.); dmiller5@ufl.edu (D.M.); thuo@ufl.edu (T.H.); mcardel@ufl.edu (M.C.); 3 Department of Psychology, Florida State University, Tallahassee, FL 32306, USA; maner@psy.fsu.edu; 4 Center for Integrative Cardiovascular and Metabolic Disease, University of Florida, Gainesville, FL 32611, USA

**Keywords:** youth, socioeconomic status, social status, eating behaviors, obesity, overweight, racial/ethnic minority, Hispanic/Latino, Hispanic American

## Abstract

Relative to other racial/ethnic groups in the United States, Hispanic American (HA) youth have higher rates of overweight and obesity. Previous work suggests that low perceived social status (SS) promotes excess caloric intake and, thereby, development of obesity. Psychological resilience may play a role in reducing adverse eating behaviors and risk for obesity. The objective of this study was to investigate whether resilience (as measured by the Connor Davidson Resilience Scale) interacts with experimentally manipulated SS to affect dietary intake among HA adolescents (*n* = 132). Using a rigged game of Monopoly (Hasbro, Inc.), participants were randomized to a high or low SS condition. Following the Monopoly game, participants consumed an *ad libitum* lunch and their dietary intake was assessed. There was a significant interaction between resilience and experimentally manipulated SS for total energy intake (*p* = 0.006), percent energy needs consumed (*p* = 0.005), and sugar intake (*p* = 0.004). For the high SS condition, for each increase in resilience score, total energy intake decreased by 7.165 ± 2.866 kcal (*p =* 0.014) and percent energy needs consumed decreased by 0.394 ± 0.153 (*p =* 0.011). In the low SS condition, sugar intake increased by 0.621 ± 0.240 g for each increase in resilience score (*p =* 0.011). After correction for multiple comparisons, the aforementioned interactions, but not simple slopes, were statistically significant.

## 1. Introduction

Through the experimental manipulation of social status (SS) in a randomized controlled trial, the current research characterizes the relationship between experimentally manipulated SS, psychological resilience, and dietary intake in Hispanic American (HA) youth. 

Relative to other racial/ethnic counterparts, HA youth are disproportionately impacted by overweight and obesity in the United States [1]. Higher body mass index (BMI) among HA adolescents has been associated with low socioeconomic status (SES) [2]. This association between low SES and obesity may be due, in part, to excess caloric intake through the consumption of energy-dense foods [3]. 

Low subjective SS, which is distinct from SES in that it reflects one’s perceived standing in society or the community, has been associated with poor physical health outcomes independent of socioeconomic status [4,5]. While low SES and low subjective SS are independently linked with increased weight, in some studies, subjective SS has been found to be a better predictor of adolescent weight status than objective SES [6,7]. During the college transition, low subjective SS has been linked with obesity. Additionally, subjective SS declines more consistently during this period of development among females and Latinos [8]. Low subjective SS is also associated with discrimination [9] and food insecurity [10], both of which are especially prevalent among racial/ethnic minorities including HA [11]. Thus, low subjective SS presents one possible factor influencing development and prevalence of obesity [12]. 

Compared to research on SES, research on the impact of subjective SS on dietary intake—particularly among groups most at risk for overweight and obesity such as HA—is relatively sparse. Few studies have investigated the relationship between subjective SS and diet. Previous studies using experimental manipulations of SS have found that individuals in a low SS condition exhibit increased intended or actual calorie consumption [13,14,15,16,17] and increases in ghrelin, an appetite-stimulating hormone [18]. Due to the possible association between low SS and increased risk for obesity, it is important to identify factors such as psychological resilience that may ameliorate this risk. 

Many definitions of psychological resilience have been put forth by different researchers and fields. Generally, psychological resilience is understood as the ability to overcome adversity and this may include thriving, flourishing, or growing above original levels of functioning despite challenges or stressful experiences [19].

Indeed, psychological resilience may serve as a link in the pathway between SES and health outcomes [20] and resilience may play a particularly strong role among racial/ethnic minorities [21]. Moreover, key components of resilience, such as perceptions of control and social support, may lessen the adverse physical health outcomes associated with SES [22]. Sense of control, for example, moderates the relationship between social class and self-reported health and wellbeing such that individuals of lower income who have a high sense of control show levels of health and wellbeing comparable to those with higher incomes [23]. The “shift and persist” model of resilience suggests that, among children with low SES, the adoption of shift and persist strategies protect them from developing chronic diseases such as cardiovascular disease in part by dampening acute physiological responses to stress [24]. Shifting includes components related to resilience such as reappraisal of stressful situations and emotion regulation. Individuals higher in shift strategies have shown lower vascular reactivity in acutely stressful tasks [24,25]. Persist strategies include resilience-related tendencies such as optimism or finding meaning in life [24], which are related to better health outcomes in daily life as well as in response to stressors [24].

Psychological resilience could serve as a protective factor that reduces the likelihood of obesity [26], particularly for those with low SS. Such protective effects could be due to the role of resilience in decreasing stress levels and resultant eating behaviors [27]. For example, psychological resilience has been shown to moderate the relationship between perceived stress and binge eating pathology [27]. Psychological resilience has also been positively associated with intake of fruits, vegetables, dietary fiber, and fish [28,29] and decreased consumption of soft drinks and takeaway food [29]. Optimism—a personality characteristic consistently reported in resilient and thriving individuals—has been associated with higher intake of fruit, vegetables, grains and lower intake of sugar [30,31]. Together, these findings suggest psychological resilience may play a role in reducing unhealthy eating behaviors and risk for obesity [26,27].

Despite the established relationship between low SS and adverse health outcomes [12,32], and between psychological resilience and eating behaviors [27,29], few studies have examined the interaction between resilience and SS in the context of dietary intake. While existing work has focused on the relationship between resilience and SES or SS in the context of other adverse health outcomes, we are not aware of any existing studies investigating whether psychological resilience dampens the harmful impact of SS on dietary intake. Applied in the context of the present study, this body of evidence suggests that psychological resilience might buffer against the adverse effects of experiencing low SS. However, research characterizing the relationship between psychological resilience, SS, and dietary intake is needed to test this model.

Through the experimental manipulation of SS in a randomized controlled trial, this study examines the relationship between psychological resilience and the acute impact of SS on dietary intake (total calories, percent of calorie needs, saturated fatty acids (SFA in grams (g)), sodium milligrams ((mg)), and sugar (g)). The authors hypothesized that psychological resilience would be associated with decreased intake of total calories, percent of calorie needs, SFA (g), sodium (mg), and sugar (g) in both the experimentally manipulated high and low status condition.

## 2. Materials and Methods

### 2.1. Participants

As previously described in other studies, potential HA participants 15 to 21 years old were recruited in North Central Florida and were screened to determine eligibility [17]. Exclusions included any severe dietary restrictions, loss or gain of more than 10 pounds in the last six months, tobacco use, birth outside of the United States, or a body mass index outside the range of 18.5–40 kg/m^2^. Those born outside of the United States were excluded in order to eliminate confounding given that recently immigrated Hispanics may have differing perceptions, risks, and experiences when compared to Hispanics born in the United States [33]. To remove participants whose subjective SS may be difficult to manipulate, participants with very low (<3) or very high (>8) subjective SS scores on the MacArthur Ladder of Subjective Social Status were excluded. 

### 2.2. Protocol

The protocol for this study was developed based on a proof of concept study [15]. The findings and methods for the primary outcomes of this larger randomized controlled trial are published elsewhere [17]; thus, the methods will focus on measures pertaining to this secondary analysis. The study was conducted according to the guidelines of the Declaration of Helsinki and approved by the Institutional Review Board of the University of Florida. Each participant was asked to fast for 12 h prior to the clinic visit and to refrain from strenuous exercise and excessive alcohol consumption for 24 h prior to the study. Physiological measures, including anthropometrics and resting metabolic rate, were assessed upon arrival. The participant was then given a standardized breakfast consisting of bottled spring water and a bacon, egg, and cheese sandwich (190 kilocalories (kcal), 9g fat, 17g car-bohydrate, 11g protein, 4g sugar, 580mg sodium, 1g fiber). After breakfast, the participant completed a series of questionnaires to assess food security, psychological resilience, and subjective SS. 

Once the participant completed the questionnaires, the research staff told the participant they would score them and return with their test results, but they simply opened up randomization envelopes. Based on randomization to either the low or high status condition, the participant was told either “I’m sorry, based on your test scores you have been given the shoe piece” (low status) or “Congratulations, based on your test scores you have been given the Rolls Royce piece” (high status).

The participant then engaged in a 40-min rigged game of Monopoly (Hasbro, Inc.) with a confederate player who occupied the opposite status condition from the participant. The rules for the low SS condition included starting the game with $1000 in the bank, collecting $100 when passing “Go”, and rolling only one die each turn. Conversely, the rules for the high status condition included starting the game with $2000 in the bank, collecting $200 when passing “Go”, rolling both dice each turn, and playing the role of the banker. Aside from these rule changes, the rest of the Monopoly game was played using standard rules of the game. During the game, the *ad libitum* buffet lunch was prepared and food weights were assessed. 

After the game, the participant was taken to an isolated room and provided an *ad libitum* buffet lunch (Table 1). The participant was left alone for 20 min to consume the lunch. Study staff weighed the foods after consumption. After the clinic visit, the participant was instructed to record their food intake via a food diary for 24-h and to wear an accelerometer for the next 24 h to measure physical activity. 

A detailed account of the status manipulation checks and main effects of the manipulation have been published previously [17]. Briefly, participants in the low SS condition had significantly fewer Monopoly resource earnings, felt significantly less powerful, and felt significantly more frustrated than those in the high SS condition [17]. 

### 2.3. Materials

#### 2.3.1. Anthropometrics

BMI was calculated by dividing height squared from weight using the formula kg/m^2^. Using a wall mounted stadiometer (Holtain Limited Harpenden, Crosswell, Crymych, UK; Veeder-Root, Elizabethtown, NC, USA), height was measured to the nearest 0.1 cm. Using a digital scale (Health O Meter 2600KL Wheelchair Scale, McCook, IL, USA), participants were weighed to the nearest 0.1 kg with shoes off.

#### 2.3.2. Food Security

Food security was assessed via a two-item screener where participants were asked to identify how often the items were true: “Within the past 12 months, I have worried whether my food would run out before I got money to buy more” and “Within the past 12 months, I couldn’t afford to eat balanced meals” [34]. Participants had the options of “never true”, “sometimes true”, or “often true”. If a participant indicated “sometime true” or “often true”, they were considered food insecure [34]. 

#### 2.3.3. Socioeconomic Status and Subjective Social Status

SES was measured via reports of household income and highest level of education. Subjective SS was measured using the MacArthur Ladder of Subjective Social Status, which has demonstrated validity and reliability in adult and adolescent populations [35,36]. The scale asks participants to rank their position in their community (or school for those <18 years). At the top of the ladder are the people who are the best off, those “with the most money, the highest amount of schooling, and the jobs that bring the most respect” while the bottom of the ladder has those who are the worst off, those with “the least money, little or no education, no jobs or jobs that no one wants or respects” [36]. Then, the individual is asked to rank their own standing on the ladder, which goes from 1 to 10, with 10 being the highest social standing in the community and 1 being the lowest.

#### 2.3.4. Psychological Resilience: Connor Davidson Resilience Scale (CD-RISC)

Psychological resilience was measured using the CD-RISC, a 25-item questionnaire assessing levels of resilience. Example items, included here with permission from the CD-RISC authors, include questions such as “I give my best effort no matter what the outcome may be” and “During times of stress/crisis, I know where to turn for help” [37]. Participants rated each item from 0 “not true at all” to 4 “true nearly all of the time” and item values were summed to produce a total score. Total scores ranged from 0 being the lowest to 100 being the highest. Higher CD-RISC scores indicate higher resilience and lower CD-RISC scores indicate lower resilience. Original factor analysis of the CD-RISC in previous research has revealed its assessment of 5 distinct factors including “personal competence, high standards, and tenacity”, “trust in one’s instincts, tolerance of negative affect, and strengthening effects of stress”, “positive acceptance of change and secure relationships”, “control”, and “spiritual influences” [37]. The CD-RISC questionnaire has been used across a large range of populations [37] and has been shown to be a reliable measure of resilience among adolescents of various races and ethnicities in many countries, including Hispanic populations [38,39]. 

#### 2.3.5. Physical Activity Energy Expenditure

Participants were fitted with an ActiGraph wGT3X-BT (ActiGraph, Pensacola, FL, USA) accelerometer, a device that measures three dimensional participant movement, to wear on their right hip during the 24 h observation period. The device was initialized at a sampling rate of 30 Hertz and collected data in 60 s epochs. Data were wear-time validated using the Choi 2011 algorithm and physical activity energy expenditure in kcal was calculated using a regression model by Ekelund et al. [40,41].

#### 2.3.6. Resting Metabolic Heartrate

Resting metabolic rate (RMR) was assessed using the validated Parvo Medics TrueOne 2400 (Sandy, UT, USA) [42,43]. Participants were asked to lie supine for 10 min and next, a 30-min assessment of RMR was completed with a hood while metabolic measurements were collected in 30 s increments. The first and last five minutes of measures were dropped and the remaining measures were averaged to calculate RMR. The value of RMR is expressed as calories burned at rest.

#### 2.3.7. Dietary Intake

The ad libitum buffet lunch included several options for food and beverage consumption totaling 1970 kcal Appendix A Table A1. Consumption of the ad libitum buffet lunch was measured in grams (g) by weighing the meal components to the nearest 0.01 g before and after the meal was provided to the participant. All consumption data were entered into the Nutrition Data System for Research (NDSR) 2017 (Nutrition Coordinating Center, University of Minnesota, USA). Variables used for analysis were total energy intake, percent of daily energy needs consumed, SFA(g), sodium (mg), and sugar (g) consumed. Percent of daily energy needs consumed was calculated using RMR, physical activity energy expenditure collected via the accelerometer, and an assumed dietary induced thermogenesis of 10% [17]. The calories consumed from the ad libitum lunch were then divided by the total energy expenditure (derived from resting metabolic rate plus energy expenditure and 10% thermic effect of food) to represent the percent of daily energy needs consumed at the lunch. 

### 2.4. Statistical Analysis

This is a secondary analysis from a randomized controlled trial assessing the effect of manipulated SS by Monopoly game on acute food intake [17]. Statistical analysis was conducted using SAS 9.4 (SAS Institute, Cary, NC, USA). The predictors of interest were psychological resilience and experimentally manipulated SS, and control variables included BMI, sex, food security, and community subjective SS. Independent and dependent variables were plotted as scatter plots to observe trends and patterns between experimentally manipulated SS, psychological resilience, and food intake. Descriptive statistics are reported as mean ± SD for continuous variables and n (%) for categorical variables (Table 2). A general linear regression model was used to estimate the association between the explanatory variables and the outcomes. Residual analyses were conducted to ensure the residuals met the model assumptions. Collinearity between the explanatory variables was also checked and the interaction terms that caused collinearity were eliminated from the model. We performed a backward model selection. Non-significant interaction terms were dropped from the model. Comparisons were adjusted for multiple comparisons using a Bonferroni correction and thus considered statistically significant at α of 0.05/5 = 0.01, given there were five outcome variables.

Additionally, we determined the direction of these relationships (positive or negative) and whether they were statistically different from zero by the general linear regression models with dummy coding of either high SS = 0 or low SS = 0 [44]. Comparisons were considered statistically significant at an α-value of 0.01.

## 3. Results

Participant demographics are shown in Table 1 for the sample overall and by SS and resilience categories. Only one of the individuals screened on the phone was excluded for having a subjective SS score outside of our range (a score of 9 on the phone screen). Of the total study sample (*n* = 133), one participant was excluded from statistical analysis due to missing CD-RISC scores. A total of 132 HA participants between the ages of 15 and 21 were included in the analysis (Table 1). Participants had a mean age of 19.1 ± 1.3 years, 60.6% were female, 74.4% were food secure, and 86.4% were college students. Our participants reported mean CD-RISC scores of 72.2 ± 10.5, which is consistent with average CD-RISC scores among college students in the United States [45,46]. Additional participant demographics are shown in Table 1. For the purposes of presenting descriptive statistics, a cutoff of 73 points, the median score on the CD-RISC from our sample, was used to categorize participants into either high or low resilience subgroups [47]; however, for all analyses, resilience was used as a continuous variable [47]. The breakdown of participant demographics by high and low SS groups, as well as by high (>73) and low (<73) resilience reveals similarly matched participant demographics for those in the high and low SS and high and low resilience groups. Appendix A Table A2 displays differences in dietary intake, our outcome variables of interest, for participants in high and low SS and high and low resilience groups.

**Table 1:** Demographics by social status (SS) condition and resilience level at baseline.

Characteristic	All (*n* = 132)	Manipulated High SS Group		Manipulated Low SS Group	
		Resilience < 73 (*n* = 26)	Resilience ≥ 73 (*n* = 38)	Resilience < 73 (*n* = 29)	Resilience ≥ 73 (*n* = 39)
Age (mean, SD)	19.1 ± 1.3	19.2 ± 1.3	19.1 ± 1.2	19.2 ± 1.0	19.0 ± 1.5
Female (*n*, %)	80 (60.6%)	17 (65.4%)	22 (57.9%)	16 (55.2%)	25 (64.1%)
BMI (mean, SD)	24.4 ± 4.1	25.6 ± 4.6	23.7 ± 4.3	24.3 ± 3.5	24.5 ± 4.0
Community SSS (*n*, % low)	37 (28.0%)	6 (23.1%)	9 (23.7%)	9 (31.0%)	13 (33.3%)
Food Security (*n*, % secure)	98 (74.2%)	20 (76.9%)	30 (78.9%)	19 (65.5%)	29 (74.4%)
Resilience	72.2 ± 10.5	60.9 ± 7.0	79.3 ± 4.9	63.5 ± 6.8	79.2 ± 6.8

Regression coefficients and *p*-values in Table 2 show the relationship between variables of interest and each of our dietary intake variables. Interactions between resilience and manipulated SS condition are also included in Table 2. There were significant interactions between resilience and experimentally manipulated SS for total energy [b ± SE: 11.270 ± 4.051 (*p* = 0.006)], percent energy needs consumed [b ± SE: 0.621 ± 0.217 (*p* = 0.005)], and sugar [b ± SE: 0.994 ± 0.341 (*p* = 0.004)]. These interactions remained significant after performing a Bonferroni control for multiple comparisons (α < 0.01). Following correction for multiple comparisons, the interaction between resilience and experimentally manipulated SS was not statistically significant (α < 0.01) for SFA [b ± SE: 0.084 ± 0.046 (*p* = 0.073)] and sodium [b ± SE: 8.905 ± 7.892 (*p* = 0.261)].

**Table 2:** Regression coefficients and *p*-values for each dietary outcome variable. *

Variable		SFA	Sodium	Sugar	Total Energy Intake	% Energy Needs Consumed
**Female**	**b ± SE**	−4.76 ± 0.73	−785.39 ± 124.47	−20.41 ± 5.37	−453.15 ± 63.88	−13.23 ± 3.42
	***p***	<0.001 *	<0.001 *	0.001 *	<0.001 *	0.053 *
**BMI**	**b ± SE**	0.05 ± 0.06	4.12 ± 10.28	−0.20 ± 0.44	0.50 ± 5.28	−0.68 ± 0.28
	***p***	0.118	0.431	0.934	0.387	0.072
**Low community SSS**	**b ± SE**	0.33 ± 0.56	101.54 ± 94.83	1.42 ± 4.09	31.59 ± 48.67	2.70 ± 2.60
	***p***	0.311	0.186	0.318	0.173	0.109
**Food Secure**	**b ± SE**	−0.92 ± 0.57	−80.15 ± 97.34	−10.29 ± 4.20	−119.41 ± 49.96	−4.63 ± 2.67
	***p***	0.229	0.583	0.039*	0.052	0.174
**Resilience**	**b ± SE**	−0.06 ± 0.03	−4.3 0 ± 5.58	−0.37 ± 0.24	−7.17 ± 2.87	−0.39 ± 0.15
	***p***	0.961	0.6353	0.365	0.845	0.739
**Low manipulated SS**	**b ± SE**	−2.97 ± 0.77	−343.32 ± 131.30	−10.06 ± 5.67	−243.97 ± 67.39	−9.83 ± 3.60
	***p***	0.136	0.486	0.242	0.265	0.460
**Female X Low SS**	**b ± SE**	3.69 ± 1.00	470.19 ± 169.85	9.46 ± 7.33	322.12 ± 87.18	13.31 ± 4.66
	***p***	<0.001 *	0.007 *	0.242	<0.001 *	0.007 *
**Resilience X Low SS**	**b ± SE**	0.08 ± 0.05	8.91 ± 7.89	0.99 ± 0.34	11.27 ± 4.05	0.62 ± 0.22
	***p***	0.073	0.261	0.004 *	0.006 *	0.005 *

* beta values (b) are unstandardized beta coefficients.

**Figure 1:**
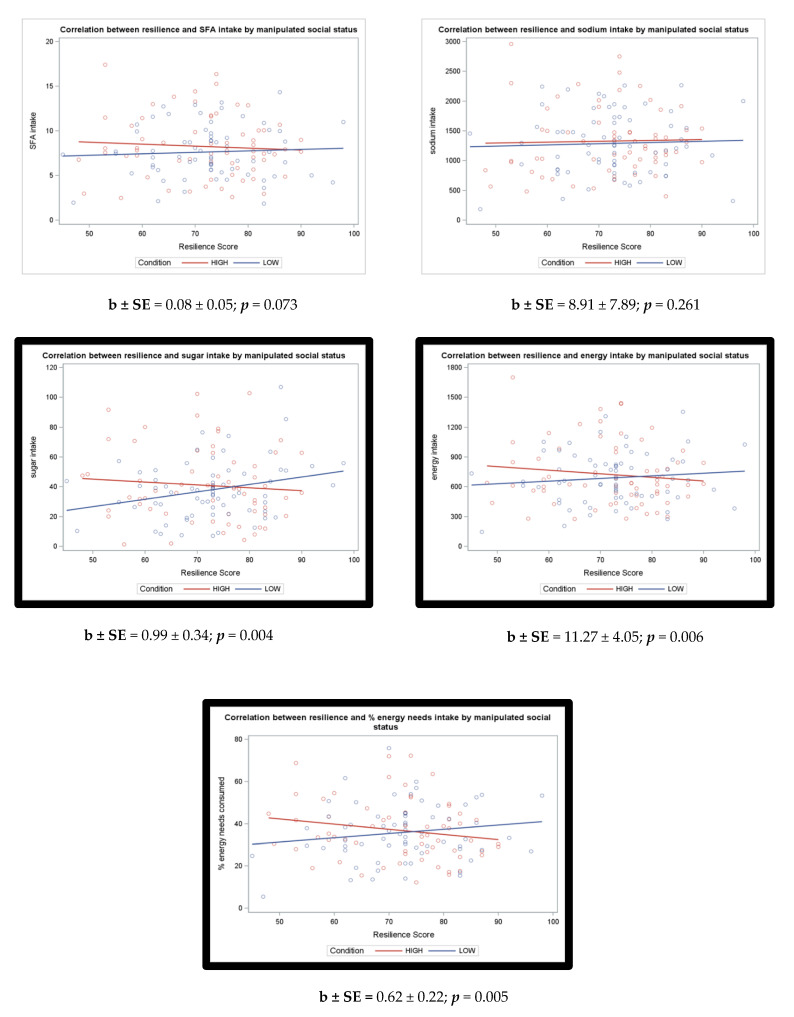
Correlation graphs and *p*-values from regression models assessing whether psychological resilience significantly moderates The relationship between high and low SS conditions and dietary intake.

Additionally, using linear regression, we tested whether the slopes were significantly different from zero (Table A3 and Figure 1). Consistent with previous research, the p-values were computed from general linear regression models with dummy coding of either high status = 0 or low status = 0 [44]. After correction of multiple comparisons using α < 0.01, simple slopes were not statistically significant (Table A3). Results from this additional analysis also indicated the direction (positive or negative) of these relationships with outcome variables. These additional tests determined the direction of the association between resilience and outcomes within each experimentally manipulated SS group more closely. Although slopes were not significant, the findings confirm the direction of the relationship seen in the scatter plots (Table A3 and Figure 1).

Our findings suggest that psychological resilience may relate differently to some measures of dietary intake in high and low SS conditions.

Literatures suggest that there would also be a negative relationship between resilience and dietary intake for each of the outcome variables such that individuals with higher resilience, when faced with low SS, would consume fewer total calories, percent of calorie needs, SFA (g), sodium (mg), and sugar (g). However, in the low SS condition, we did not observe this. There was a positive (although non-significant) relationship between resilience and each of the outcome variables. Of note, sugar intake increased by 0.621 ± 0.240 g for each increase in CD-RISC score (*p =* 0.0108), however this slope was not significant after Bonferroni correction for multiple comparisons.

## 4. Discussion

We characterized the relationship between psychological resilience, experimentally manipulated SS, and dietary intake in a randomized controlled trial of experimentally manipulated SS. We tested whether psychological resilience significantly moderated the relationship between experimentally manipulated SS condition and dietary intake.

Significant interactions between manipulated SS condition and psychological resilience emerged for three of five outcome variables: sugar, total energy intake, and percent energy needs consumed. This suggests that the relationship between psychological resilience and some dietary intake variables may differ based on experimentally manipulated SS condition. However, none of the simple slopes in our analysis were significant and thus more research is needed to confirm the direction of these relationships.

Within the high SS condition, Bonferroni corrected simple slopes were not significant (Table A3). Thus, we do not have statistically significant evidence that psychological resilience protected against high dietary intake in the high SS condition. There are several possible explanations for this finding. One such explanation may relate to the nature of psychological resilience. Psychological resilience is understood as recovering or flourishing in the face of adversity. Those in the high SS condition did not experience SS-related adversity like those in the low SS condition did. Thus, it is possible that when removed from the context of adversity, psychological resilience does not have an impact on dietary intake. Another possible explanation for the lack of a statistically significant relationship between psychological resilience and dietary intake in the high SS condition could simply be due to low power. Some of the simple slopes were close to our Bonferroni corrected significance threshold of 0.01. For example, total caloric intake (*p =* 0.014) and percent of energy needs consumed (*p =* 0.011) were nearly significantly negatively associated with psychological resilience. With only 64 participants in the high SS condition, it is possible that we did not have the power to detect significant effects. Some of these relationships—perhaps especially those between psychological resilience and caloric intake—may be significant in a larger sample. Future research is needed clarify whether these relationships exist along with their potential directions.

While simple slopes were nonsignificant in our study, the overall pattern is in line with the authors’ a priori hypothesis of a negative association between psychological resilience and some forms of dietary intake (particularly caloric intake). This may reflect the idea that individuals with greater psychological resilience may be able to maximize the experience of a high SS environment resulting in decreased caloric intake, and, perhaps, risk for obesity. Thus, this is an area ripe for future research.

Simple slopes suggest that psychological resilience also did not significantly serve as a protective factor against high dietary intake in the low SS condition (Table A3). Experimental manipulations of SS by our group and others have shown that the mere experience of perceived low SS is enough to increase intended and actual food consumption [13,14,15,16]. Thus, for individuals in the low SS condition, it is possible that psychological resilience did not protect against high intake of dietary outcome variables because the effects of the low SS condition overpowered the effects of psychological resilience. Of note, simple slopes from our analyses indicate that increased psychological resilience may even be associated with increased dietary intake in low SS conditions, particularly for sugar intake (*p* = 0.011), which showed the strongest positive, although non-significant, association between these variables. The number of participants in the low SS condition (*n* = 68) was relatively small and it is possible that significant relationships would emerge with a larger number of participants. Thus, more research is needed to confirm these associations and clarify the directionality of these relationships.

There are several possible explanations for the surprising positive trend between psychological resilience and dietary intake variables in the low SS condition. Though the relationship was not significant, one possible explanation for higher resilience scores relating to greater dietary intake of sugar in the low status condition could be that individuals with higher resilience in the low status condition may be eating highly palatable, energy-dense foods to try to restore their mood. This explanation aligns well with robust self-esteem literature showing that individuals with high self-esteem are more motivated than those with low self-esteem to restore positive mood after a negative event, such as failure or rejection [48], which may have occurred in the low social status condition. Indeed, self-esteem is predictive of trait resilience and many of the questions in the CD-RISC scale appear to tap into facets of self-esteem (e.g., “I am able to adapt when changes occur” and “I take pride in my achievements”) [37,49,50,51]. Additionally, original factor analysis of the CD-RISC revealed both “personal competence, high standards, and tenacity” and “control”, both of which are relevant to self-esteem, to be distinct factors of the scale [37]. Finally, eating to improve one’s mood is a commonly reported coping strategy [52]. Studies in humans and animals have linked sugar intake with dopamine levels and activation of reward circuitry in the brain which may explain its role in enhanced mood [53,54,55]. Eating as a mood regulation behavior has also been correlated with being female, having less education, and having overweight [52]. Thus, it is possible that after playing a rigged game in which they perceive they likely have no chance of winning, those in the low status condition with higher resilience may have turned to energy-dense foods high in sugar to replenish their positive mood.

The phenomena of skin-deep resilience and John Henryism described among studies of Black youth and adults may also help explain the paradoxical (albeit statistically nonsignificant) finding of increased dietary intake, particularly sugar, with increased resilience in the low SS condition. Skin-deep resilience is a phenomenon seen among Black adolescents and adults from disadvantaged backgrounds who, despite apparent positive psychosocial functioning and high resilience, suffer poor physical health, such as high allostatic load [56,57]. A longitudinal study of rural African American youth found that, despite their low SES position, these youth showed fewer instances of adverse behaviors in young adulthood. Consistent with the literature, self-control during youth predicted psychological resilience and apparent positive outcomes—such as academic achievement—in young adulthood in this cohort. However, an unexpected, positive relationship was found between self-control and adverse physical health outcomes in this group: Black youth of low SES with higher levels of self-control were found to have higher risk of cardiometabolic disease (measured via obesity, blood pressure, and stress hormones). That is, while high self-control was associated with positive outcomes associated with psychological resilience in young adulthood (e.g., low substance use and depressive symptomology), high self-control was associated with poorer health outcomes (e.g., greater cardiometabolic risk) in the same cohort [57,58]. Like their non-Hispanic Black counterparts, HA adolescents occupy a minority position in the United States and may share adverse experiences such as discrimination [56]. Thus, the positive relationship between resilience and sugar intake in the low status condition may reveal an instance of skin-deep resilience. 

John Henryism is marked by an active, high-effort, and persistent method of coping with psychosocial and environmental demands via hard work and determination to succeed [59]. While adaptive in the presence of adequate economic and social resources John Henryism is related to adverse outcomes among those with fewer resources including higher blood pressure and autonomic arousal [59] as well as metabolic syndrome [60]. Participants utilizing John Henryism strategies in the low SS condition may have high resilience scores, but still experience increased dietary intake and, thus, adverse physical outcomes. However, we did not specifically test for John Henryism coping strategies in our participants, and therefore our interpretation is speculative. Importantly, skin-deep resilience and John Henryism have only been investigated among Black adolescents, which may not directly relate to our sample of HA adolescents, as research suggests important distinctions in the experience of non-Black HA and non-Hispanic Black populations. Additionally, these racial/ethnic identities are not mutually exclusive. More research is needed to understand whether non-Black HA populations experience skin-deep resilience and John Henryism. Future research should also acknowledge that non-Black HA and Black populations are not the same and there is likely important individual variability in these experiences. Still, this may be an important avenue for future research investigating the relationship between SS, race/ethnicity, and adverse eating behaviors.

This research has important implications for clinical and intervention research on psychological resilience as well as those addressing eating behaviors and aimed at decreasing overweight/obesity, particularly among HA adolescents. For example, results from our study suggest that interventions aimed at increasing psychological resilience may not be effective for decreasing adverse eating behaviors and/or overweight/obesity among HA adolescents with low SS. However, more research is necessary to confirm this relationship. It is important for clinicians and researchers alike to consider the complex and nuanced relationships between SS, psychological resilience, and eating behaviors.

### 4.1. Strengths and Limitations

Generalizability of this study is limited due to our inclusion of an entirely HA sample; however, the exclusion of non-HA individuals and Hispanics born outside of the United States limited the introduction of confounding variables that can exist between race/ethnicity and SS in the United States. Additionally, as a laboratory experiment on acute dietary intake, our study may not have captured many of the real-life and more chronic factors affecting SS among HA adolescents, including discrimination, which may impact dietary intake over the long-term [9]. Despite this, the randomized controlled trial design permitted an experimental manipulation of SS. Additionally, we were able to investigate the potential moderating effects of psychological resilience on the relationship between SS and acute eating behaviors and among a large sample of HA adolescents—a historically understudied population in the literature. Dietary intake was measured rigorously in controlled conditions that permitted objective assessments of consumption and physical activity energy expenditure data were also calculated from an objective measure. Finally, the CD-RISC is a well-validated questionnaire with adequate psychometric properties that assesses five different aspects of resilience, primarily related to individual psychological resilience [37,38,39,61]. This conceptualization of resilience using the CD-RISC may be limited as recent research has emphasized that resilience—particularly resilience related to health outcomes among racially/ethnically minoritized populations—is a multi-level (i.e., individual, interpersonal, and neighborhood-level) and dynamic process [62]. Thus, future research measuring resilience among racially/ethnically minoritized groups may be better served by incorporating a malleable, state-level model of resilience, which includes a greater emphasis on interpersonal components such as social support as well as neighborhood social capital.

### 4.2. Future Directions

This research is an important first step in investigating the relationship between psychological resilience and dietary intake in the face of subjective SS-related adversity. This study suggests that psychological resilience may interact with SS to impact dietary intake of sugar and calories. Future research that explores this relationship using different measures of resilience may shed light on the nature of these associations. Additionally, replications and extensions of this work with larger samples, among other racial/ethnic groups, and with different age groups would extend our knowledge of the roles of SS and psychological resilience on eating behavior in other populations. It would be useful for future studies to include a measure of resilience both before and after SS manipulation, which would allow researchers to account for within person changes in resilience that could be attributable to the SS manipulation. Longitudinal studies could help categorize the dynamic or stable nature of this relationship over time. More research is needed to fully understand the effects of psychological resilience among diverse racial/ethnic groups and how these effects might depend on context.

## 5. Conclusions

This study suggests that psychological resilience may interact with SS to impact dietary intake of sugar and calories. Contrary to our hypothesis, we did not find evidence that psychological resilience may buffer against the adverse effects of experimentally manipulated low SS on acute dietary intake for HA. Results from this study help characterize the extent to which psychological resilience influences eating behavior and risk for obesity, particularly as this relates to SS. Greater knowledge about the relationship between psychological resilience, status, and dietary intake could inform resilience-building interventions and weight loss interventions and other public health initiatives to help treat and/or prevent overweight and obesity, which now impacts over 34% of the United States population and disproportionally impacts HAs [63].

## Data Availability

Individual participant data will be available (including data dictionaries). In particular, individual participant data that underlie the results reported in this article will be shared after deidentification (text, tables, figures, and appendices). Other documents that will be available include the study protocol. Upon request, investigator(s) whose proposed use of the data would need to be approved by an independent review committee identified for this purpose and could be allowed access for individual participant data meta-analysis. Proposals may be submitted up to 36 months following article publication. After 36 months, the data will be available in our university’s data warehouse but without investigator support other than deposited metadata.

## Appendix A

**Table A1:** Nutritional Composition of *Ad Libitum* Lunch Foods.

Food	Weight (g)	Calories (kcal)	Fat (g)	Saturated Fat (g)	Carbohydrate (g)	Protein (g)	Sugar (g)	Sodium (mg)	Fiber (g)
Meat Lasagna	283	410	19	8	40	20	6	750	3
Green Beans	411	70	0	0	14	3.5	7	1015	7
Macaroni and Cheese	227	280	14	4.5	30	7	1	800	2
Doritos Nacho Cheese	88	420	24	3	48	6	0	630	3
Classic Applesauce	113	90	0	0	20	0	17	20	2
Fruit Cup	113	70	0	0	18	0	17	0	1
Oreo Cookies	68	320	13	3.5	49	3	27	270	1
Bottled Water	500	0	0	0	0	0	0	0	0
Cola	355	160	0	0	43	0	43	35	0
Pink Lemonade	355	150	0	0	42	0	40	50	0

**Table A2:** Dietary Intake by SS Condition and Resilience Level.

Dietary Intake Variable	All (*n* = 132)	Manipulated High SS Group		Manipulated Low SS Group	
		Resilience < 73 (*n* = 26)	Resilience ≥ 73 (*n* = 38)	Resilience < 73 (*n* = 29)	Resilience ≥ 73 (*n* = 39)
Total SFA, g (mean, SD)	7.9 ± 3.2	8.5 ± 3.9	8.1 ± 3.2	7.7 ± 3.0	7.6 ± 2.9
Sodium, mg (mean, SD)	1307 ± 541	1306 ± 660	1342 ± 505	1320 ± 551	1265 ± 496
Total sugar, g (mean, SD)	39.3 ± 21.9	44.3 ± 26.7	38.6 ± 22.8	34.1 ± 17.3	40.6 ± 20.4
Total energy, kcal (mean, SD)	707 ± 289	774 ± 364	691 ± 289	692 ± 281	688 ± 240
% energy needs, (mean, SD)	36.3 ± 13.7	39.1 ± 15.0	35.4 ± 13.5	34.5 ± 15.0	36.8 ± 12.2
Total SFA, g (mean, SD)	7.9 ± 3.2	8.5 ± 3.9	8.1 ± 3.2	7.7 ± 3.0	7.6 ± 2.9

**Table A3:** The effect of resilience on dietary outcome variables within high or low experimentally manipulated SS conditions.

		Effect of Resilience			
Outcome	SS Condition	Coefficient	Standard Error	t Value	*p*-Value *
SFA (g)	High	−0.056	0.033	−1.70	0.092
	Low	0.028	0.033	0.86	0.393
Sodium (mg)	High	−4.302	5.584	−0.77	0.443
	Low	4.604	5.560	0.83	0.409
Sugar (g)	High	−0.373	0.241	−1.55	0.125
	Low	0.621	0.240	2.59	0.011
Energy (kcal)	High	−7.165	2.866	−2.50	0.014
	Low	4.105	2.854	1.44	0.153
% Energy needs	High	−0.394	0.153	−2.57	0.011
	Low	0.228	0.153	1.49	0.138

* *p*-values are used to test whether the slope is significantly different from zero. They are computed from the general linear regression models with dummy coding of either high SS = 0 or low SS = 0 [44] After correction for multiple comparisons, values were considered to be statistically significant at *p* < 0.01.
